# Supplementary material for: Social deprivation and SARS-CoV-2 testing: a population-based analysis in a highly contrasted southern France region
Source: Front Public Health. 2023 May 12;11:1162711. doi: 10.3389/fpubh.2023.1162711 (PMC10213643; doi:10.3389/fpubh.2023.1162711)
Supplement: Supplementary file 1 [file Data_Sheet_1.PDF]

Social deprivation and SARS-CoV-2 testing: a population-based analysis in a highly contrasted Southern France region

Jordi Landier [1], Léa Bassez [1], Marc-Karim Bendiane [1], Pascal Chaud [2], Florian Franke [2], Steve Nauleau [3], Fabrice Danjou [3], Philippe Malfait [2], Stanislas Rebaudet [1,4], Jean Gaudart[5]

1. *Aix Marseille Univ, IRD, INSERM, SESSTIM, Aix Marseille Institute of Public Health, ISSPAM, Marseille, France*
2. *Santé Publique France Cellule régionale Paca-Corse, Marseille, France*
3. *Agence Régionale de la Santé Provence Alpes Côte d'Azur, Marseille, France*
4. *Hôpital Européen Marseille, Marseille, France*
5. *Aix Marseille Univ, APHM, Inserm, IRD, SESSTIM, ISSPAM, Hop Timone, BioSTIC, Marseille, France*

Supplementary figures and results

1 Supplementary material and methods

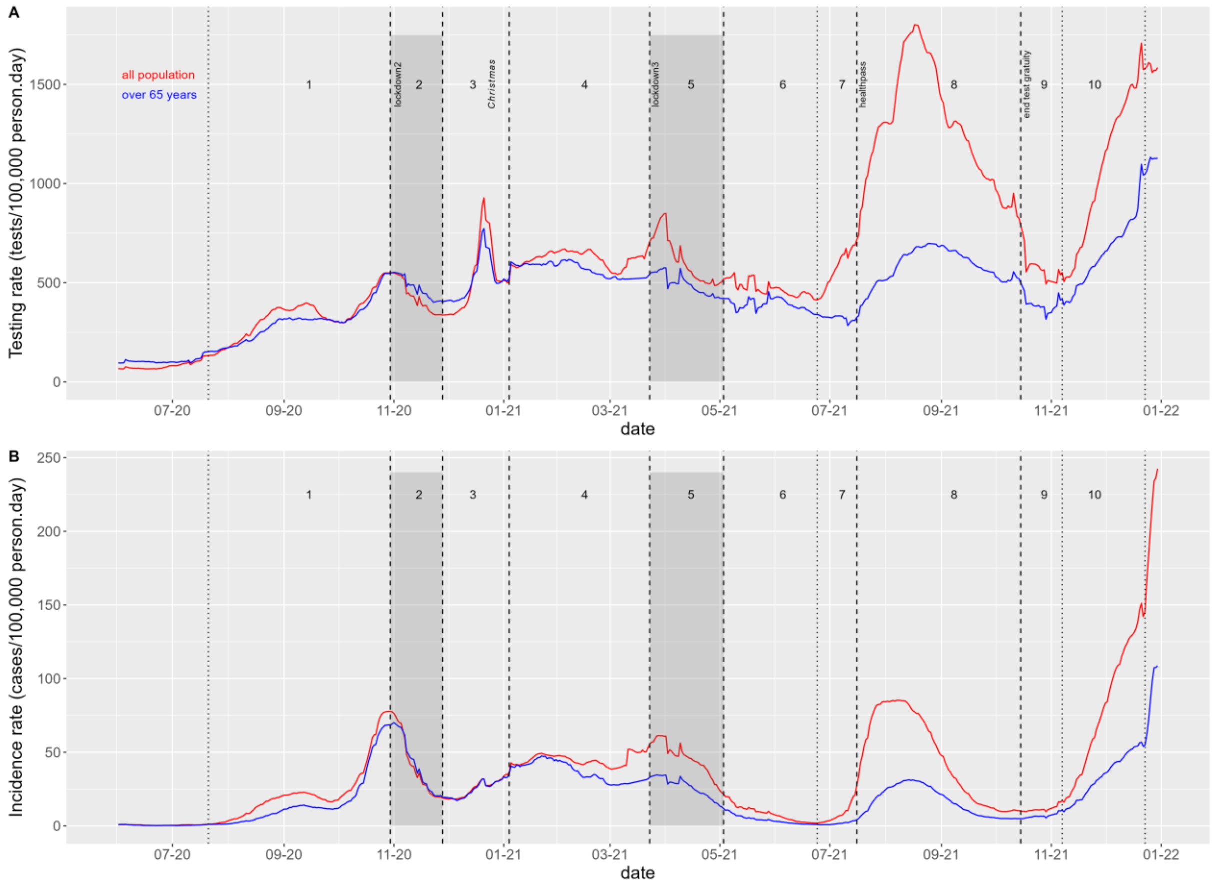

Figure S1: Daily evolution of SARS-CoV-2/COVID-19 (A) testing and (B) incidence rates in the Provence Alpes Cote d'Azur (PACA) region in the general (red) and ≥65-years-old (blue) populations.

Table S1: Description of the ten epidemic periods of the COVID-19 epidemic in France considered for Provence Alpes Côte d'Azur (PACA) regional analysis between 22 July 2020 and 22 December 2021, i.e. from the onset of the second wave to the onset of omicron variant wave.

Period n°	Start and end dates	Starting Event	Total tests	Mean Testing rate (tests per 100,000 person.days)	Total cases	Mean Incidence rate (cases per 100,000 person.days)	Description
1	22/07/2020 to 29/10/2020	Incidence starts to increase again (wave 2)	1,653,352	9,954	115,348	694.45	Wave 2 rising
2	30/10/2020 to 27/11/2020	Lockdown date	636,072	13,205	62,924	1306.32	Lockdown 2
3	28/11/2020 to 03/01/2021	1st restrictions lifted	968,744	15,763	46,956	764.05	Christmas
4	04/01/2021 to 23/03/2021	End of Christmas holidays	2,483,119	18,924	179,562	1368.42	Wave 3 rising
5	24/03/2021 to 02/05/2021	Lockdown date	1,240,047	18,664	92,356	1390.07	Lockdown 3
6	03/05/2021 to 22/06/2021	1st restrictions lifted	1,217,022	14,367	22,660	267.50	Wave 3 falling
7	23/06/2021 to 15/07/2021	local incidence minimum (wave 4)	646,405	16,920	8,384	219.46	Wave 4 rising
8	16/07/2021 to 14/10/2021	Decree instituting mandatory healthpass	5,828,291	38,560	214,360	1418.19	Wave 4 healthpass
9	15/10/2021 to 07/11/2021	End of convenience testing gratuity for unvaccinated population	698,027	17,510	14,103	353.78	End of test gratuity
10	08/11/2021 to 22/12/2021	Onset of wave 5 (delta variant)	2,440,882	32,656	181,676	2430.62	Wave 5 Delta

Table S2: Spatial unit ("IRIS") covariate data sources

Database name/type	Date of publication	Source	URL
**Permanent equipment database 2020**	12/07/2021	INSEE	https://www.insee.fr/fr/statistiques/3568629?sommaire=3568656&q=bpe+2020
**Census database**	09/12/2020	INSEE	https://www.insee.fr/fr/statistiques/4515565?sommaire=4516122&q=recensement+2017#consulter
**Available income database 2017**	29/12/2020	INSEE	https://www.insee.fr/fr/statistiques/4479212#consulter
**Housing database 2017**	09/12/2020	INSEE	https://www.insee.fr/fr/statistiques/4515532?sommaire=4516107&q=base+logement#dictionnaire
**European deprivation index (EDI)**	2015	MapInMed	https://anticipe.eu/plateformes/MAPinMED
**French deprivation index**	01/04/2019	INSERM	https://public.opendatasoft.com/explore/dataset/indice-de-defavorisation-sociale-fdep-par-iris/information/?flg=fr&q=croix&location=13,50.67628,3.13308&basemap=jawg.streets
**IRIS map (shapefile)**		French Government	https://www.data.gouv.fr/en/datasets/decoupage-iris-combine-aux-limites-municipales-openstreetmap/
**Localized potential access (LPA)**	02/03/2020	DREES	https://drees2-sgsocialgouv.opendatasoft.com/explore/dataset/530_l-accessibilite-potentielle-localisee-apl/information/

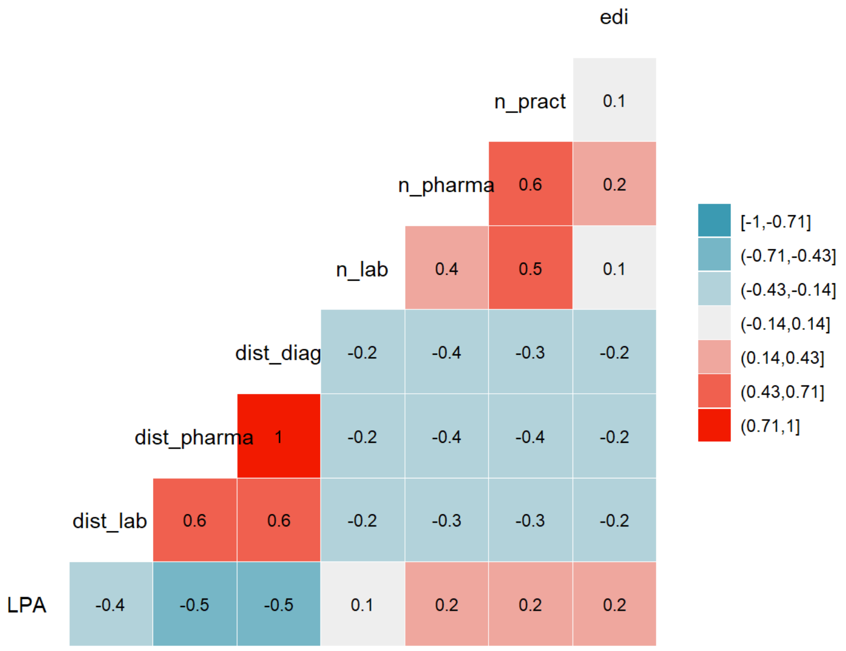

Figure S2: Spearman cross correlation matrix between health access variables. LPA= localized potential accessibility, i.e. number of general practitioner consults available per person and per year (calculated at municipal level). dist_lab = distance to the nearest laboratory performing biological exams. dist_pharma = distance to the nearest pharmacy. dist_diag = distance to the nearest equipment, combining laboratory and pharmacy. n_lab = number of laboratories in IRIS. n_pharma = number of pharmacies in IRIS. n_pract = number of primary healthcare practitioners in IRIS. edi = European deprivation index in IRIS (showing limited overall correlation with accessibility)

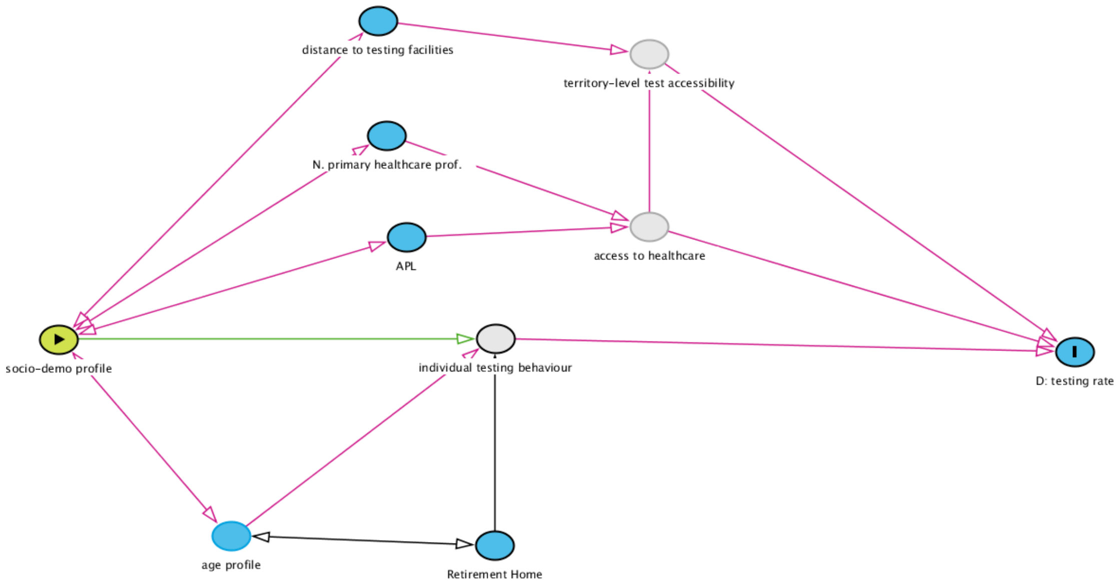

Figure S3: Directed acyclic graph used to define the relationship between socio-demographic/deprivation profiles and SARS-CoV-2 testing rate in Provence Alpes Côte d'Azur region.

2 Supplementary results

2.1 Regional distribution of IRIS descriptive socio-demographic and health access variables

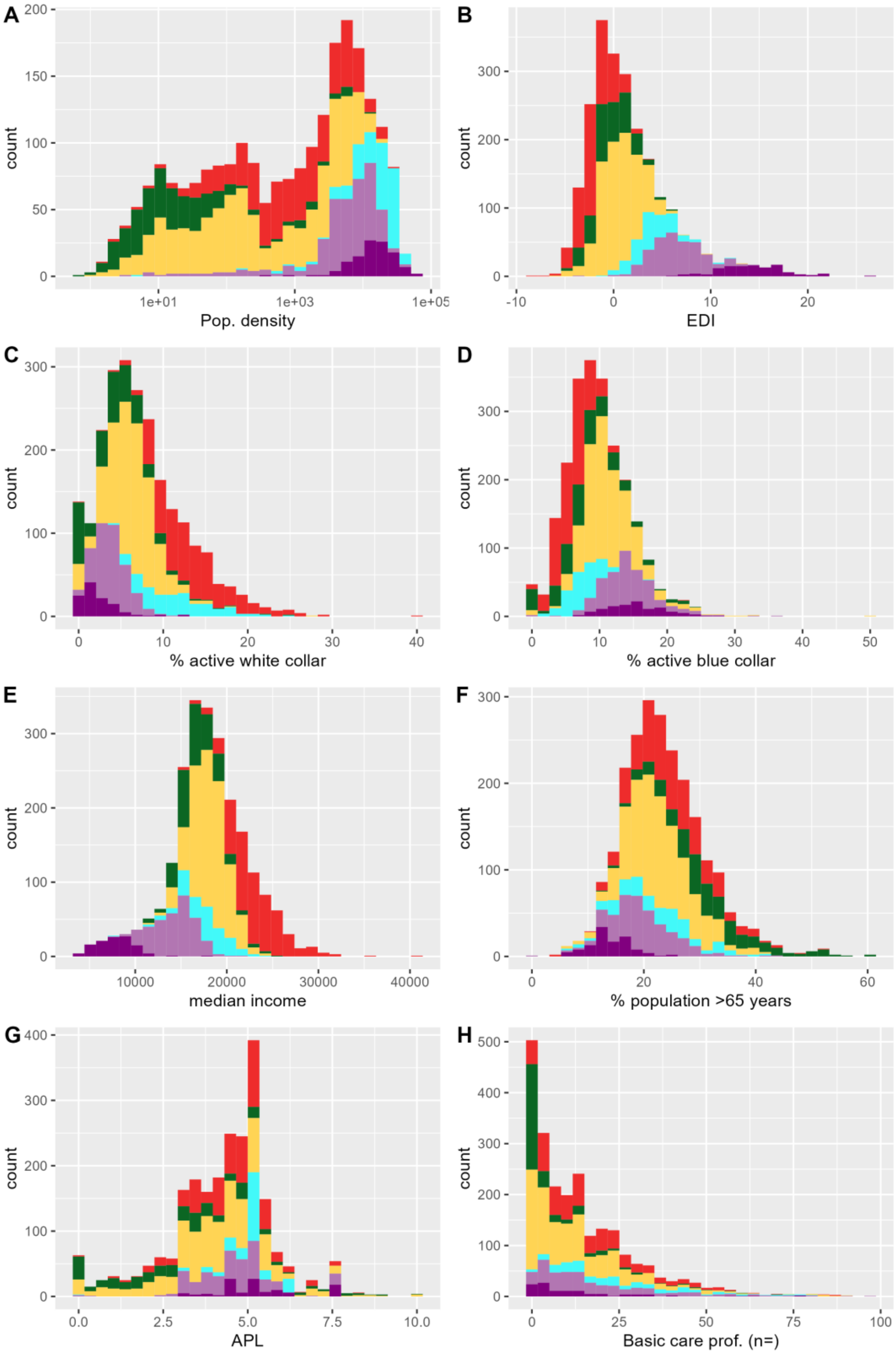

Figure S4: Histograms of socio-demographic profile variables and health access variables, by profile. Socio-demographic profiles: (1) “privileged” (red); (2) “remote” (green); (3) “intermediate” (yellow); (4) “downtown” (cyan); (5) “deprived” (light purple); (6) “very deprived” (dark purple).

2.2 Univariate regression results for period 1

Table S3: univariate results for period 1 (model including municipality-level random intercept and s(x,y) spatial autocorrelation adjustment)

Type	Variable	Category	TRR	95% confidence interval	p-value	gcv	dev.expl
access to health-care	LPA	spline	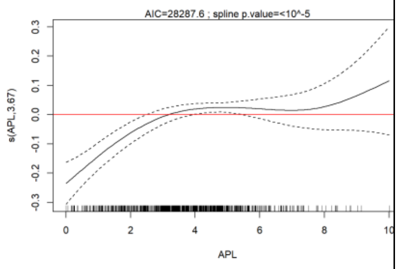		$<10^{-6}$	3778.0	74.3
	N pharmacies	0 (ref)	2.72	(ref)		3784.9	74.4
		n=1	1.03	1.01 1.06	0.009		
		n=2	1.05	1.02 1.08	0.0016		
		n>=3	1.14	1.09 1.20	$<10^{-6}$		
	N basic healthcare professionals	spline	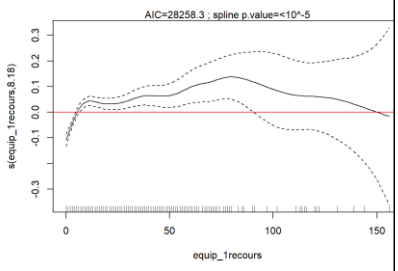		$<10^{-6}$	3791.5	74.6
	Distance to the nearest laboratory	spline	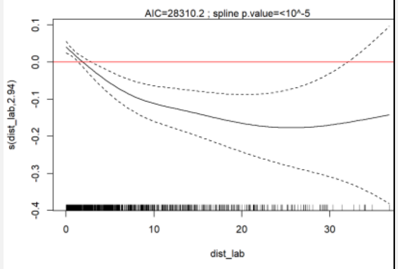		0.000007	3778.9	73.9
	Distance to the nearest pharmacy	spline	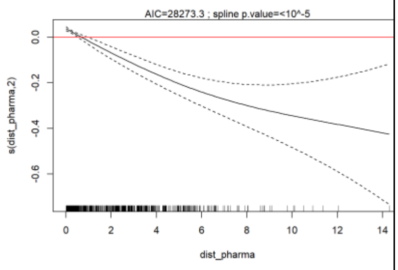		$<10^{-6}$	3763.5	74.1
age	Age profile	Families	2.72	ref		3832.3	75.7

		Balanced	1.12	1.09<br>1.15	$<10^{-6}$		
		Older adults	1.18	1.14<br>1.22	$<10^{-6}$		
		Young adults	1.08	1.04<br>1.12	0.00012<br>7		
	Percentag<br>e of<br>population<br>aged >65<br>years	spline	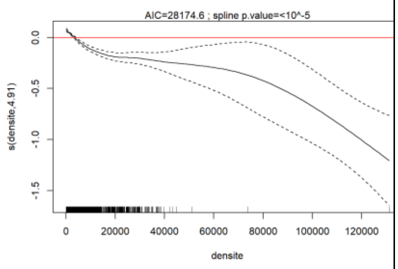		$<10^{-6}$	3818.<br>4	75.4
	retirement<br>home	No	2.72	(ref)		3751.<br>2	73.9
		yes	1.10	1.08-1.13	$<10^{-6}$		
Socio-<br>demo-<br>graphic	EDI	spline	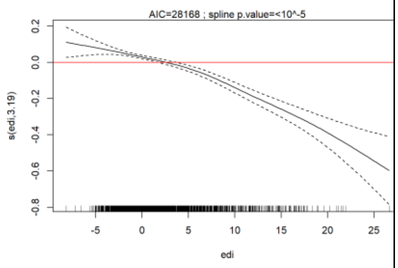		$<10^{-6}$	3890.<br>6	76.8
	National<br>EDI<br>quintile	Q1 (ref)	2.72	(ref)		3853.<br>17	75.4
		Q2	0.98	0.94-1.02	0.33979<br>1		
		Q3	0.95	0.91-1.00	0.03016<br>4		
		Q4	0.95	0.91-0.99	0.02297<br>4		
		Q5	0.89	0.85-0.92	$<10^{-6}$		
	pop.<br>density	spline	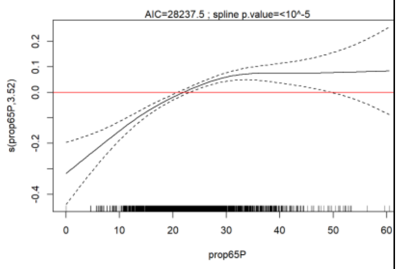		$<10^{-6}$	3867.<br>0	76.8
	SD profile	1.privileged	2.72	(ref)		3850.<br>5	75.9
		2.remote	0.89	0.85-0.93	0.00000<br>2		
		3.intermediate	0.93	0.90-0.96	0.00000<br>3		
		4.downtown	0.95	0.90-0.99	0.01598<br>5		

		5.deprived	0.87	0.84-0.90	$<10^{-6}$		
		6.very_deprived	0.73	0.69-0.77	$<10^{-6}$		

TRR, testing rate ratio; 95%-CI, 95%-confidence interval; LPA, Localized potential accessibility to healthcare; EDI, European deprivation index; SD, socio-demographic

2.3 Multivariate, all periods, splines

2.3.1 LPA splines

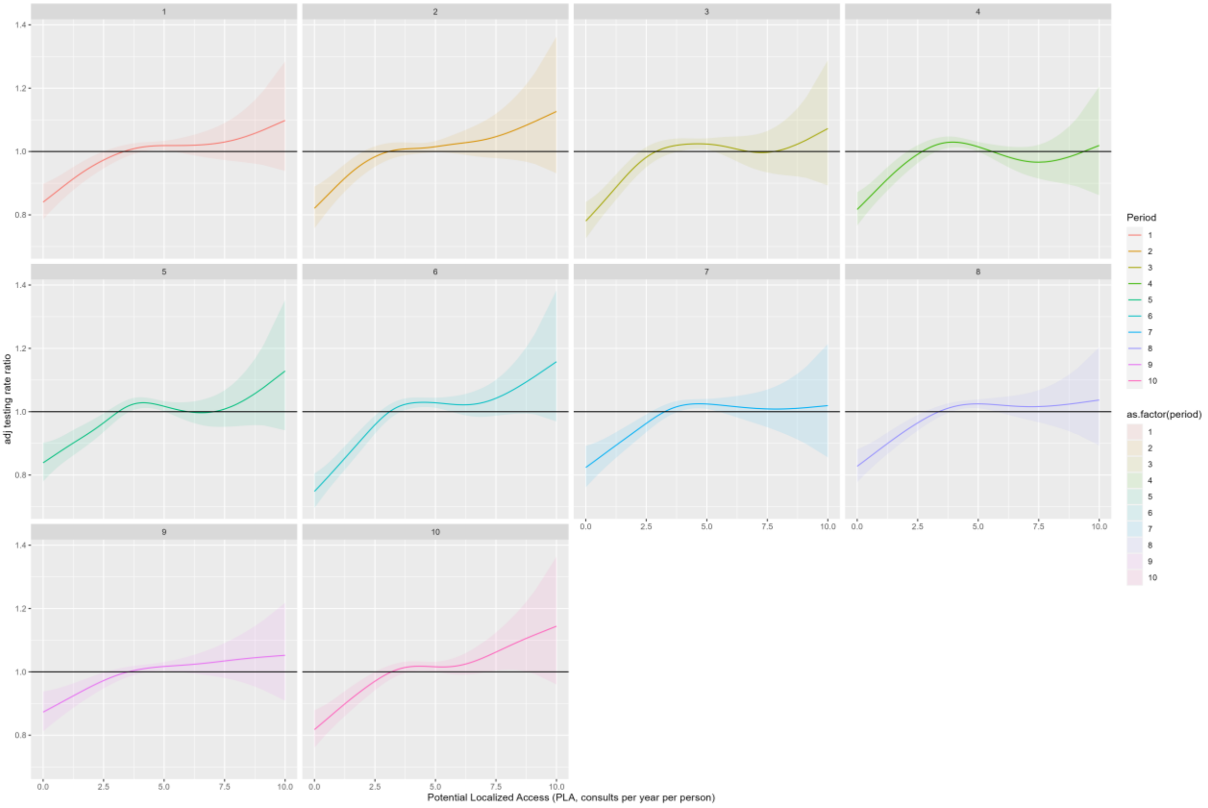

Figure S5: Non-linear effect of localized potential accessibility to healthcare (LPA) for periods 1 to 10 (panels).

2.3.2 Number of basic healthcare professionals slines

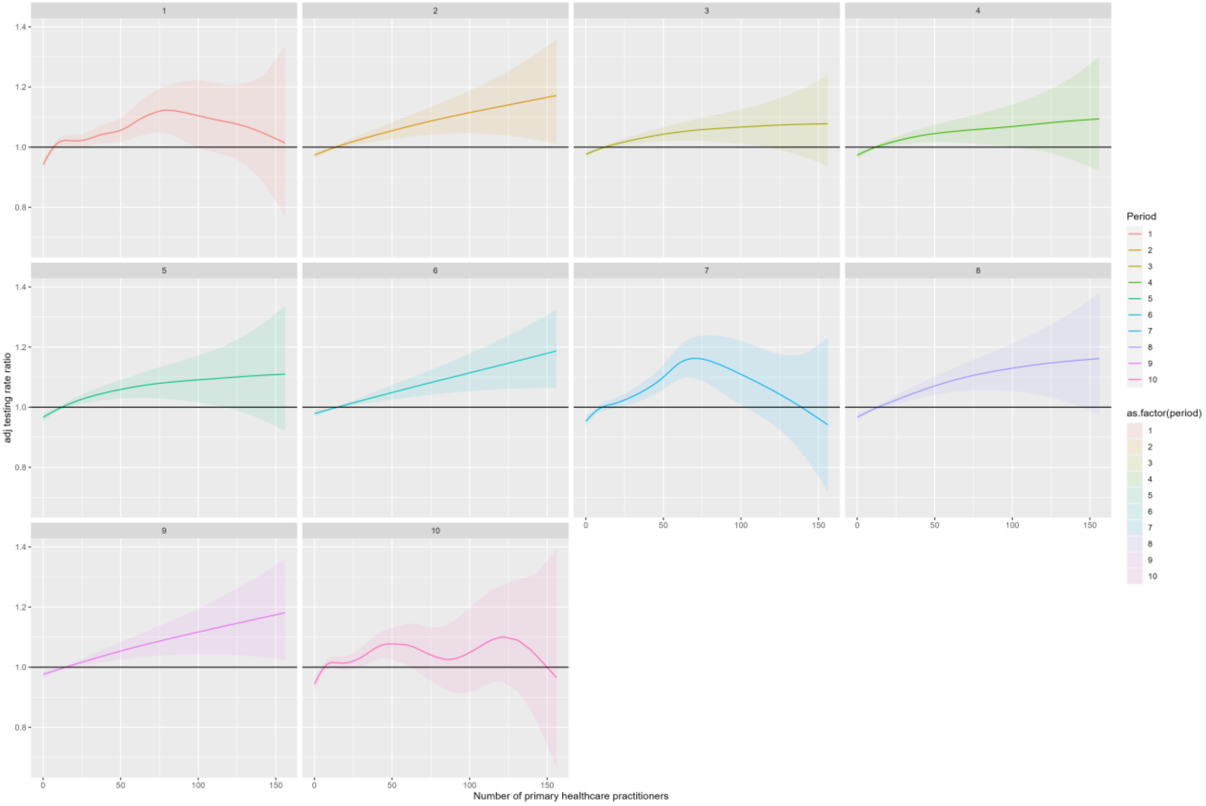

Figure S6: Non-linear effect of the number of basic healthcare professionals for periods 1 to 10 (panels).

2.4 Multivariate, all periods, sensitivity analysis

2.4.1 Replacing municipality-level LPA with distance to the nearest pharmacy

Multivariate analysis performed replacing localized potential accessibility to healthcare (LPA) with distance to the nearest pharmacy, all other variables remaining identical.

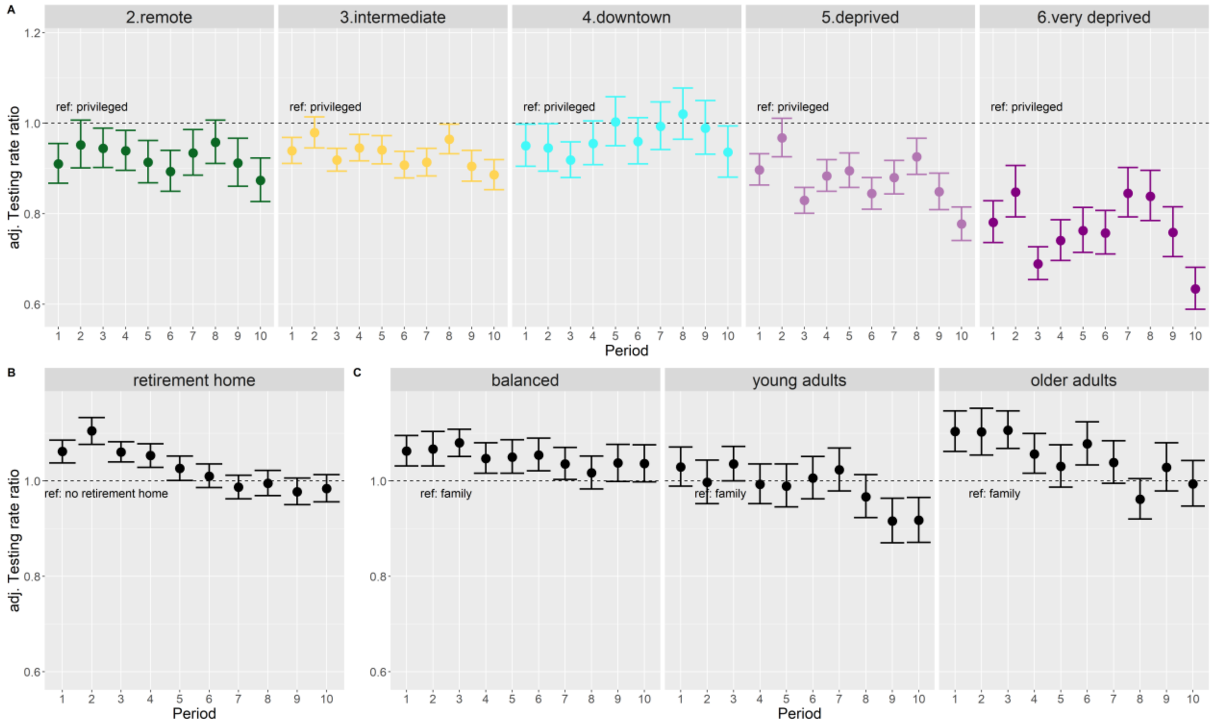

Figure S7: Combined forest plots for all periods substituting distance to the nearest pharmacy to replace localized potential accessibility to healthcare (LPA). One multivariate regression by period, all models combined for the 3 variables.

2.4.2 Replacing socio-demographic profiles with EDI values

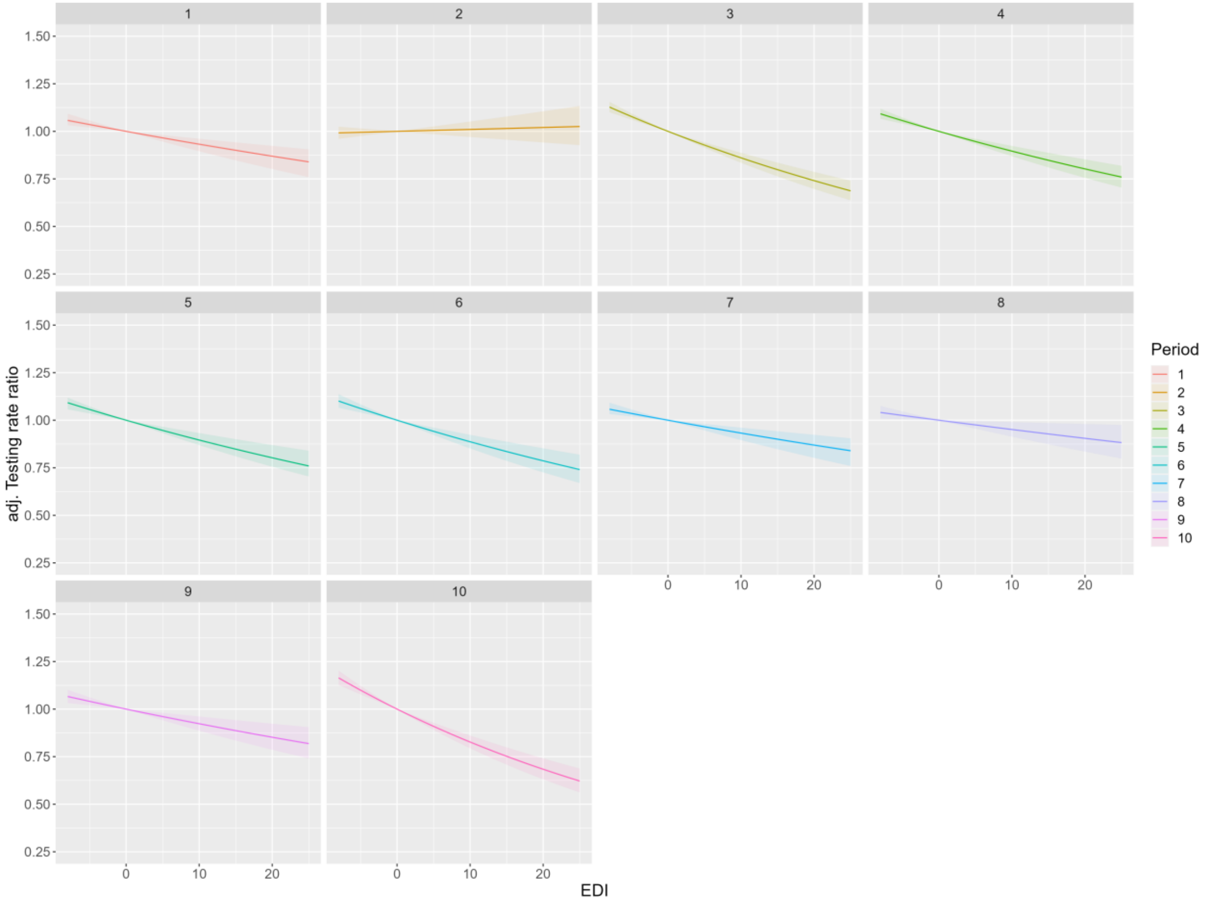

Figure S8: Adjusted Testing Rate Ratio (aTRR) by European deprivation index (EDI) value for each period, assuming an exponential effect of EDI on testing rate ratio (TRR) (i.e. a linear effect of +1 unit of EDI on log(TRR)). Multivariate negative binomial regression model including adjustments for localized potential accessibility to healthcare (LPA), number of primary care professionals, presence of a retirement home, proportion of the population above 65 years of age, population density, with a municipality-level random intercept and a Gaussian kriging smoother of the geographical coordinates of each IRIS.

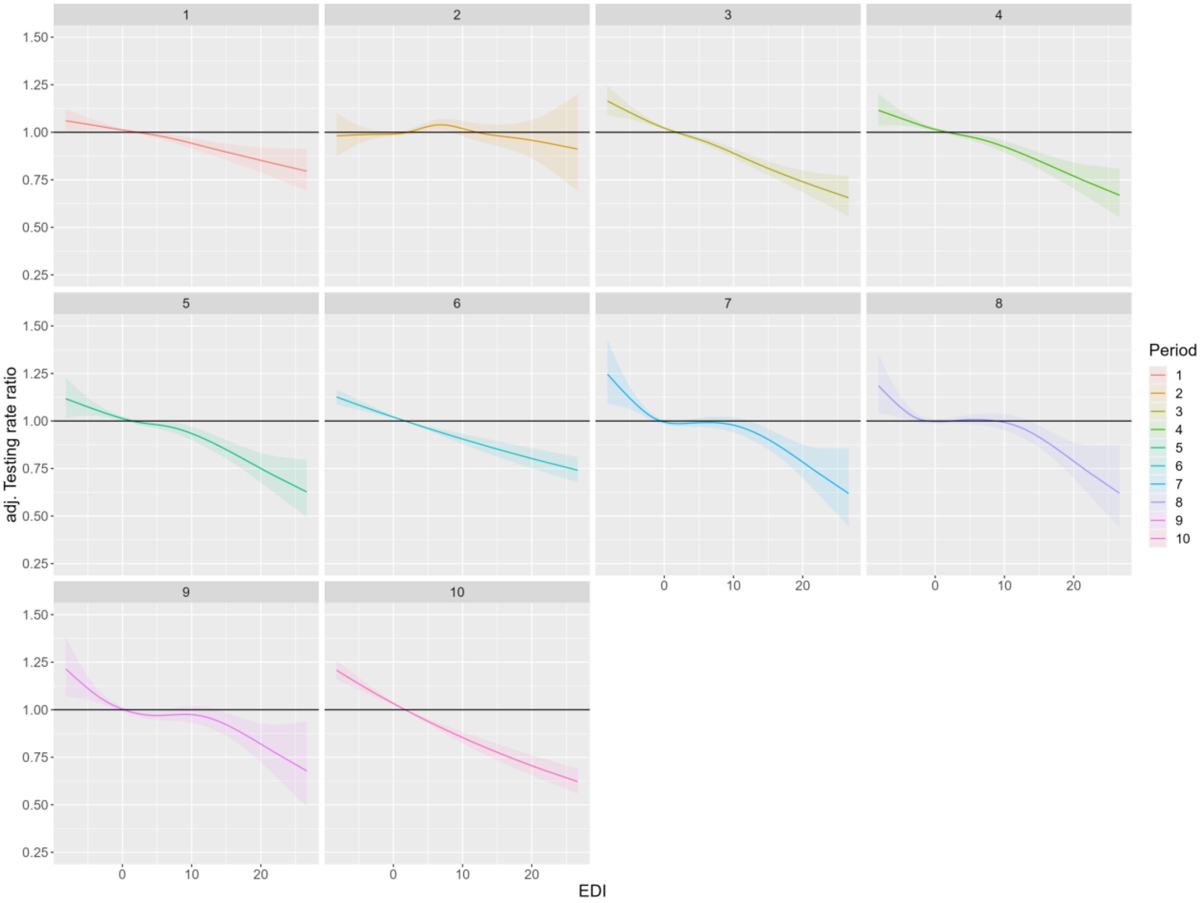

Figure S9: adjusted Testing rate ratio (aTRR) by European deprivation index (EDI) value for each period, assuming a non-linear effect of EDI on testing rate ratio (TRR) (spline). Multivariate negative binomial regression model including adjustments for localized potential accessibility to healthcare (LPA), number of primary care professionals, presence of a retirement home, proportion of the population above 65 years of age, population density, with a municipality-level random intercept and a Gaussian kriging smoother of the geographical coordinates of each IRIS.

2.5 IRIS socio-demographic profile versus national deprivation index quintiles

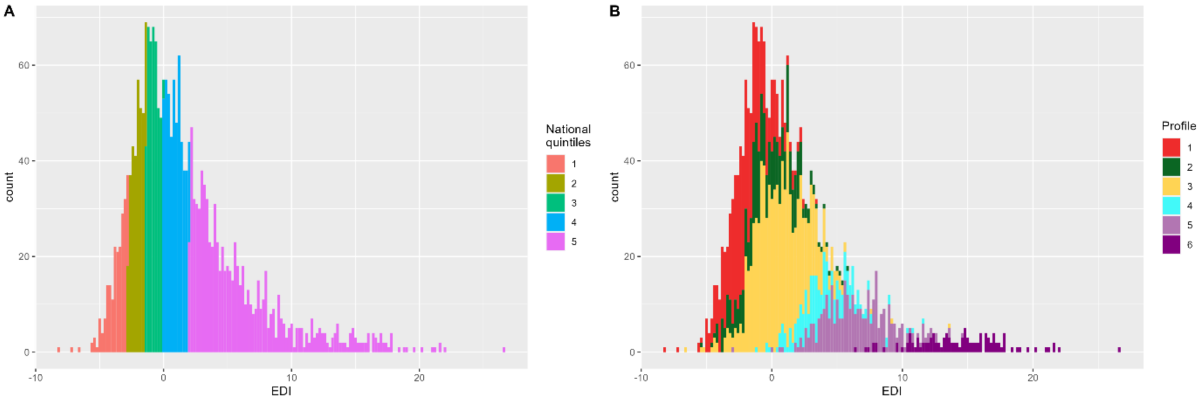

Figure S10: Distribution of European Deprivation Index (EDI) value for 2306 analysed IRIS. (A) by national EDI quintile; (B) by socio-demographic profile as defined in this study. Socio-demographic profiles: (1) "privileged" (red); (2) "remote" (green); (3) "intermediate" (yellow); (4) "downtown" (cyan); (5) "deprived" (light purple); (6) "very deprived" (dark purple).

The regional analysis of European Deprivation Index (EDI) using national quintiles would not bring sufficient precision. Indeed, of 2306 residential IRIS with >30 inhabitants, 36.51% (n=842) correspond to 5th/highest national EDI quintile. Only 9.15% (n=211) are in the first quintile, 14.01% (n=323) in the second,17.91% (n=413) in the third, and 22.42% (n=517) in the fourth (Figure S4A). The fifth quintile actually covers 4 different sociodemographic profiles in the region, corresponding to very contrasted features: "very deprived" (urban, very dense); "deprived" (urban corresponding to both neighbourhoods in large metropolitan areas and smaller industrial towns for example); "downtown" (very dense, urban, with young adults and a significant proportion of white-collar workers); "intermediate" (suburban to rural areas) (Figure S4B).
